# Habitat Use Across the Geographic Range of *Limenitis reducta* Reveals Stronger Constraints at the Northern Edge

**DOI:** 10.1002/ece3.74265

**Published:** 2026-09-03

**Authors:** Julius Kaiser, Heiko Hinneberg, Thomas K. Gottschalk

**Affiliations:** ^1^ University of Applied Forest Sciences Rottenburg Rottenburg am Neckar Germany; ^2^ Regierungspräsidium Tübingen, Referat 56: Naturschutz und Landschaftspflege Tübingen Germany

**Keywords:** host plant selection, light availability, *Limenitis reducta*, microclimate tracking, niche conservatism, northern edge populations

## Abstract

Species abundance typically declines toward the edges of geographic ranges, while their conservation relevance increases. Habitat studies therefore often focus on only small parts of species' ranges, potentially biasing our understanding of ecological niches. Here, we compared habitat use of the Southern White Admiral (*Limenitis reducta*) between populations at the northern and near the southern range edge to assess whether and how habitat associations differ across the species' distribution range. The Swabian Jura represents the northern range edge, while the Turkish Aegean region lies near the southern range edge within the Mediterranean core distribution. We systematically surveyed larval stages in both regions. We compared host plant species, host plant height, site exposition, solar radiation duration at host plants, and shading by neighboring trees. In addition, the height of the larvae above ground was recorded. Host plant species differed between regions but belonged to the genus *Lonicera* in both. The species was associated with relatively well‐sunlit sites in both regions, reflected by preferences for host plants receiving high solar radiation and low shading by neighboring trees. These associations were markedly stronger at the northern range edge. Larvae were found higher above ground at the northern range edge, and species occurrence was positively associated with host plant height in both regions. Regional differences in habitat associations indicate differences in realized microhabitat use between regions with contrasting macroclimatic conditions. The stronger association with highly sun‐exposed habitats at the northern range edge is consistent with the hypothesis that habitat use is more strongly constrained under cooler climatic conditions than near the southern range edge. These findings highlight the importance of considering multiple parts of a species' geographic range when assessing habitat associations and developing conservation strategies under changing climatic conditions.

## Graphical abstract

We compared larval habitat use of the Southern White Admiral (*Limenitis reducta*) between populations at the northern range edge in Germany and near the southern range edge in the Turkish Aegean region. Habitat associations with solar radiation, shading, host plant height and slope exposition differed between regions, with markedly stronger selection for highly sun‐exposed habitats at the northern range edge. Our findings demonstrate that habitat use varies across the species' geographic range and highlight the importance of considering regional variation when studying species' niches and developing conservation strategies.

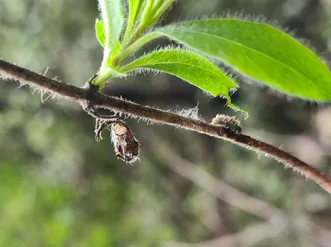

## 1 Introduction

Species often experience different environmental conditions across their geographic ranges, which can lead to variation in habitat use and ecological requirements across their distribution ranges. Range‐edge populations are typically characterized by lower densities and are less stable than populations in the core range, where environmental conditions are assumed to be near the species' ecological optimum (Thomas et al. 2001; Parmesan and Yohe 2003; Martin et al. 2021; Dederichs et al. 2024). As a consequence, habitat use at range margins may differ from that observed in the core range, potentially leading to incomplete descriptions of species' ecological niches when studies cover only small parts of a species' distribution range.

In the context of species conservation, this biogeographical variation in habitat association is likely to be particularly relevant for forest‐dwelling butterflies, which have declined across Europe despite an overall increase in forest cover (FAO 2020; Warren et al. 2021). Comprehensive data on their habitat requirements are needed to better understand their distribution patterns and to implement effective habitat management measures (Dennis 2010; Graser et al. 2023). Unfortunately, such data are rarely available because research efforts often focus on relatively small parts of species' distribution ranges, for example regions where the target species is of high conservation concern. These regions are often located at the edges of species' distribution ranges (Hampe and Petit 2005; Lesica and Allendorf 1995). Since these peripheral populations often occur under distinct climatic and habitat conditions, studying them in isolation may lead to an incomplete description of species' niches and, as a result, potentially to incorrect conclusions regarding their conservation. Evidence for distinct patterns of habitat use in range edge populations has been reported in several butterfly species. For example, Martin et al. (2021) reported that females of *Lycaena dispar* in range edge populations were more generalist and oviposited under a broader range of microhabitats than females in core populations. Thomas et al. (1998) demonstrated that *Maculinea arion* is largely confined to warm south‐facing slopes near its northern range limits, whereas preferences for particular slope orientations are less clear in warmer regions. Wallis De Vries (2001) showed that *Melitaea cinxia* overwinters mainly in standing dead vegetation on the Isle of Wight but predominantly in short vegetation in Finland and explained these regional differences by the prevailing macroclimate. Standing dead vegetation buffers fluctuations in moisture and temperature under the mild, humid and largely snow‐free winters of western Europe, whereas continuous snow cover in Finland provides insulation against harsh winter conditions. In brief, previous evidence suggests that studying species' habitat associations in different parts of the distribution range can greatly improve the understanding of habitat requirements.

Understanding regional differences in the habitat association of pre‐imaginal butterfly life stages is particularly important, because the egg, larval, and pupal stages account for most of the butterfly life cycle and are the most sensitive to environmental changes (Pollard 1979; Warren et al. 1986; Radchuk et al. 2012; Mulé et al. 2017; Hinneberg et al. 2025). This is especially important for range edge populations, often characterized by smaller population sizes due to sub‐optimal habitat conditions, reduced genetic diversity, and limited resource availability. Consequently, range edge populations often have a higher extinction risk compared with core populations (Rochat et al. 2017; Martin et al. 2021; Dederichs et al. 2024).

The Southern White Admiral (*Limenitis reducta*) is a medium‐sized nymphalid butterfly with a distribution range from northern France to Cyprus/Israel and from the Iberian Peninsula to Iran and eastern Turkey (Kudrna et al. 2011). The core area of the species' geographic distribution range is in the Mediterranean region, where it is widespread and locally occurs in relatively high numbers (Bence and Richaud 2020; Leraut 2016; Pamperis 2009). At its northern range edge in Central Europe, the species has strongly declined, for example in France (UICN Comité Français 2012), Germany (Musche et al. 2025), Switzerland (Wermeille et al. 2014) and Austria (Zulka 2005) or has become extinct (Czech Republic; Šumpich and Liška 2018). Its decline has been associated with the abandonment of clear‐cutting and traditional forest management techniques (Warren et al. 2021; Hinneberg et al. 2025).

At its northern range limit in Germany, the species is “critically endangered” (Musche et al. 2025) with the last populations found in the Swabian Jura, Baden‐Württemberg. These last German *Limenitis reducta* populations are strongly associated with open forest habitats such as windthrows, clear‐cuts or south‐facing forest margins (Hermann 2007; Hinneberg et al. 2022, 2023). Its population decline in Central Europe has motivated detailed ecological studies, including oviposition preferences and habitat associations (Hinneberg et al. 2022, 2025). Conversely, no comprehensive studies have yet been conducted in the species' core range or near its southern range edge in the Mediterranean region, that is, only descriptions of macrohabitats are available from these regions (e.g., Hesselbarth et al. 1995; Stefanescu and Jubany 2003; Benyamini 2022). In the Mediterranean, the species is predominantly reported from forested habitats (Stefanescu and Jubany 2003), especially riparian forests, calcareous woodlands, semi‐open forest edges, and heterogeneous bushy habitats (Hesselbarth et al. 1995; Pamperis 2009) but oviposition sites have not yet been characterized in detail. While different host plant species of the genus *Lonicera* have been described from the Mediterranean region (Tolman and Lewington 2012), *Lonicera xylosteum* is the only available host plant in most Central European forests. The species' phenology also differs between the southernmost populations, where up to three generations occur per year (Hesselbarth et al. 1995) and the northern range edge, where it is typically univoltine (Hinneberg et al. 2023). Similarly in both regions, the third instar of the last larval generation per year builds a leaf shelter, a so‐called hibernaculum, in August/September.

Based on data from the Swabian Jura (northern range edge) and the Turkish Aegean region (core population near the southern range edge), our study aims to compare the habitat association of *Limenitis reducta* between regions of different climatic conditions. We hypothesized that the species' clear preference for well‐sunlit larval habitats (cf. Hinneberg et al. 2022) is more pronounced at its northern range edge compared with the Mediterranean region, where the warmer macroclimate may allow the use of a wider range of habitats.

## 2 Materials and Methods

### 2.1 Study Area

**FIGURE 1:**
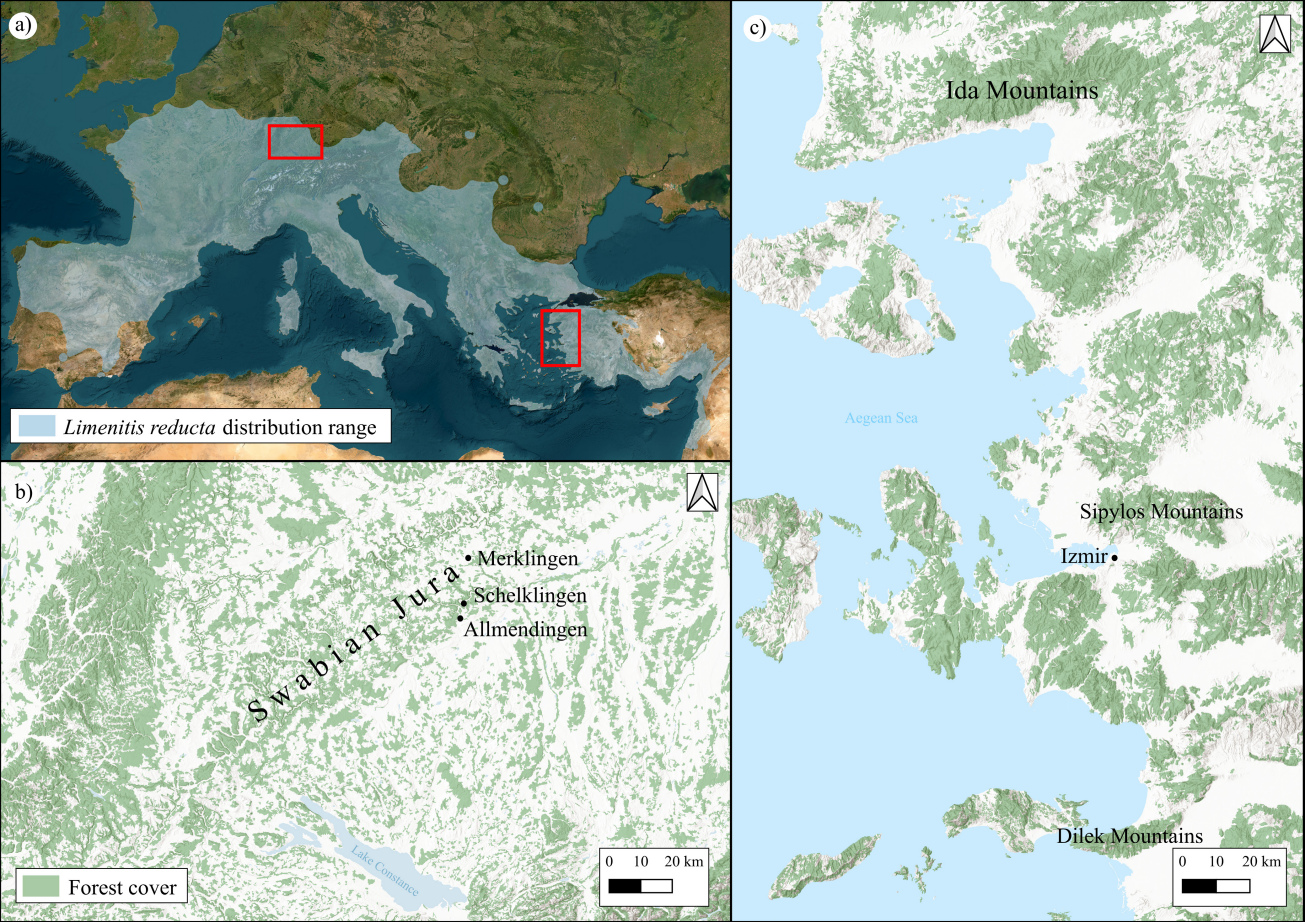
Distribution range of *Limenitis reducta* (a) showing the locations of the study areas in the Swabian Jura (Germany) (b) and the Turkish Aegean region (c). The approximate current distribution range of *Limenitis reducta* is taken from the IUCN Red List of Threatened Species (IUCN 2014, 2025). Forest cover data were derived from CORINE Land Cover (European Environment Agency 2019). The distribution range depicted here is based on current and historic records. In Germany, the current northern range edge runs further south (Reinhardt et al. 2020).

The study was conducted in two regions: (1) the Swabian Jura, representing the species' northern range edge and (2) the Turkish Aegean region, representing a region near the species' southern range edge but still within its core distribution range (Figure 1). The Swabian Jura (48.0°–49.0° N, 8.5°–10.6° E) in southern Germany is a low mountain range (400–1016 m a.s.l.) characterized by temperate climate with humid conditions throughout the year and moderately warm summers and cool winters. The mean annual temperature is 6.7°C and mean annual precipitation ranges between 750 and 1050 mm. Rainfall decreases toward the southeastern plateau of the Swabian Jura (Jooß 2013). The natural vegetation is dominated by calcareous beech forests. *Lonicera* shrubs, particularly *Lonicera xylosteum* , are generally abundant in the mountain range (Sebald et al. 1996). *Limenitis reducta* is mainly found in the southern part of the Swabian Jura, predominantly in managed forests. Data were collected from three study areas near Allmendingen, Schelklingen and Merklingen.

**FIGURE 2:**
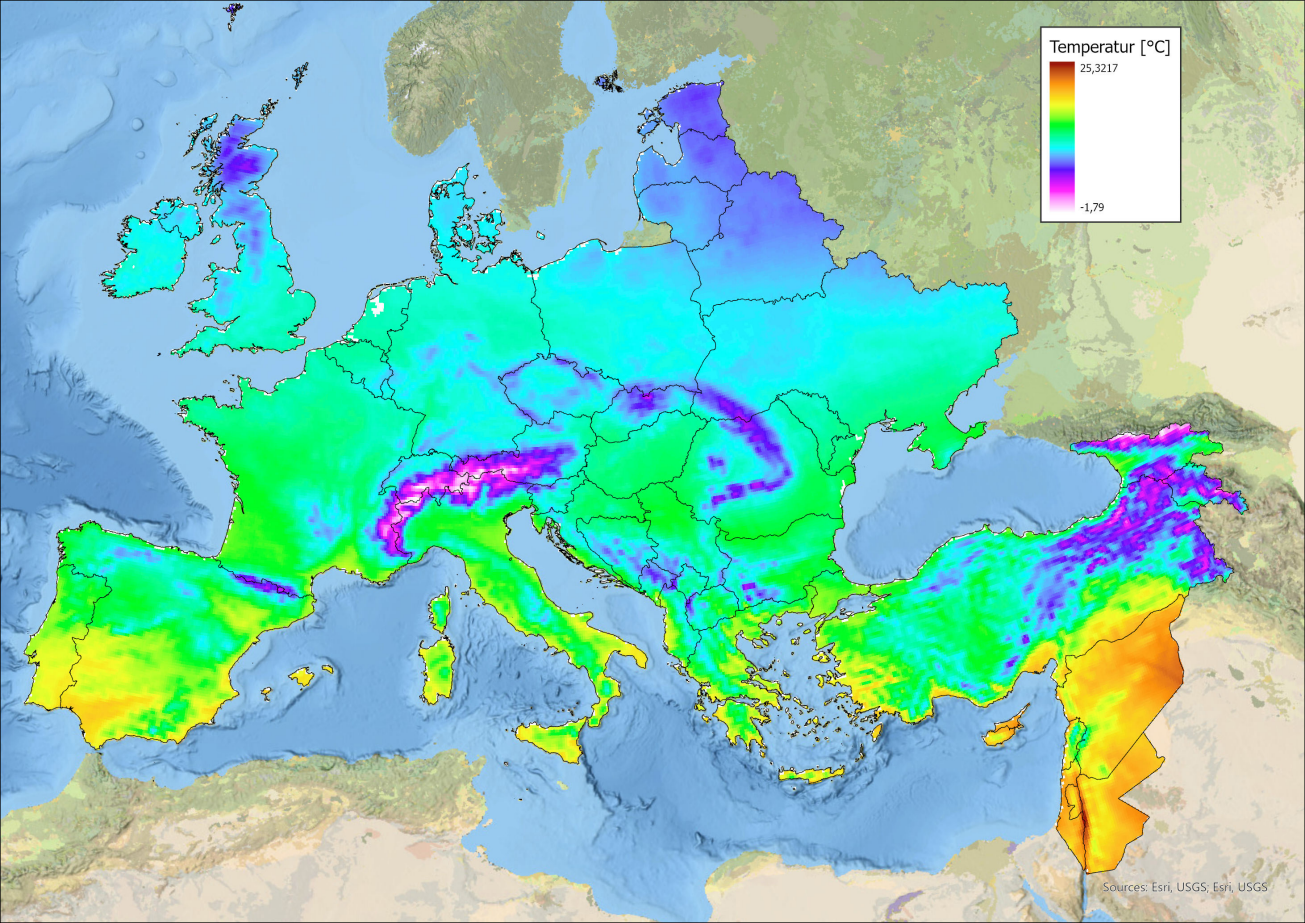
Mean annual temperature (°C) between 2016 and 2025 across Europe and the Mediterranean region. Temperature data were derived from ERA5‐Land reanalysis (Copernicus Climate Change Service 2022).

The Turkish Aegean region (36° N–39.8° N, 26° E–30.5° E) is characterized by a hot‐summer Mediterranean climate with dry, hot summers and mild, humid winters. The mean annual temperature is 16.4°C and mean annual precipitation ranges between 500 and 1100 mm (Mersin et al. 2022; Figure 2). The vegetation is dominated by deciduous oak forests and Mediterranean sclerophyllous woodlands (Mayer and Aksoy 1986). According to the habitat descriptions of Bergmeier et al. (2018) and our field observations, the larval host plants of *Limenitis reducta* (genus *Lonicera*) in the study area are *Lonicera etrusca* , *Lonicera implexa*, and the non‐native *Lonicera japonica* . As many forests in the Turkish Aegean region have been heavily affected by forestry and overgrazing, they often have little or no shrub layer (Şekercioğlu et al. 2011). Therefore, we selected forested areas with high structural diversity for this study. These are mainly located within or near national parks and nature reserves in the southern Ida, Sypilos and Dilek Mountains.

A substantial part of the study in the Ida Mountains focused on forest areas north of the Darıdere Natural Park. In this area, *Lonicera* plants grow on steep slopes, among large rock formations, and alongside streams and riverbanks. In the Sipylos Mountains, both closed‐canopy forests and forest margins/open woodlands were examined. The forest structure in the forested areas of the Sipylos Mountains was characterized by a closed canopy and dense shrub layer. Vegetation in adjacent open areas was significantly affected by wild horse grazing. In these habitats, climbing *Lonicera* species persist alongside thorny plants that protect them from browsing. The Dilek Mountains are characterized by structurally diverse vegetation. The combination of shrub vegetation and dense tree cover on steep slopes results in a multilayered forest structure.

### 2.2 Data Collection

**FIGURE 3:**
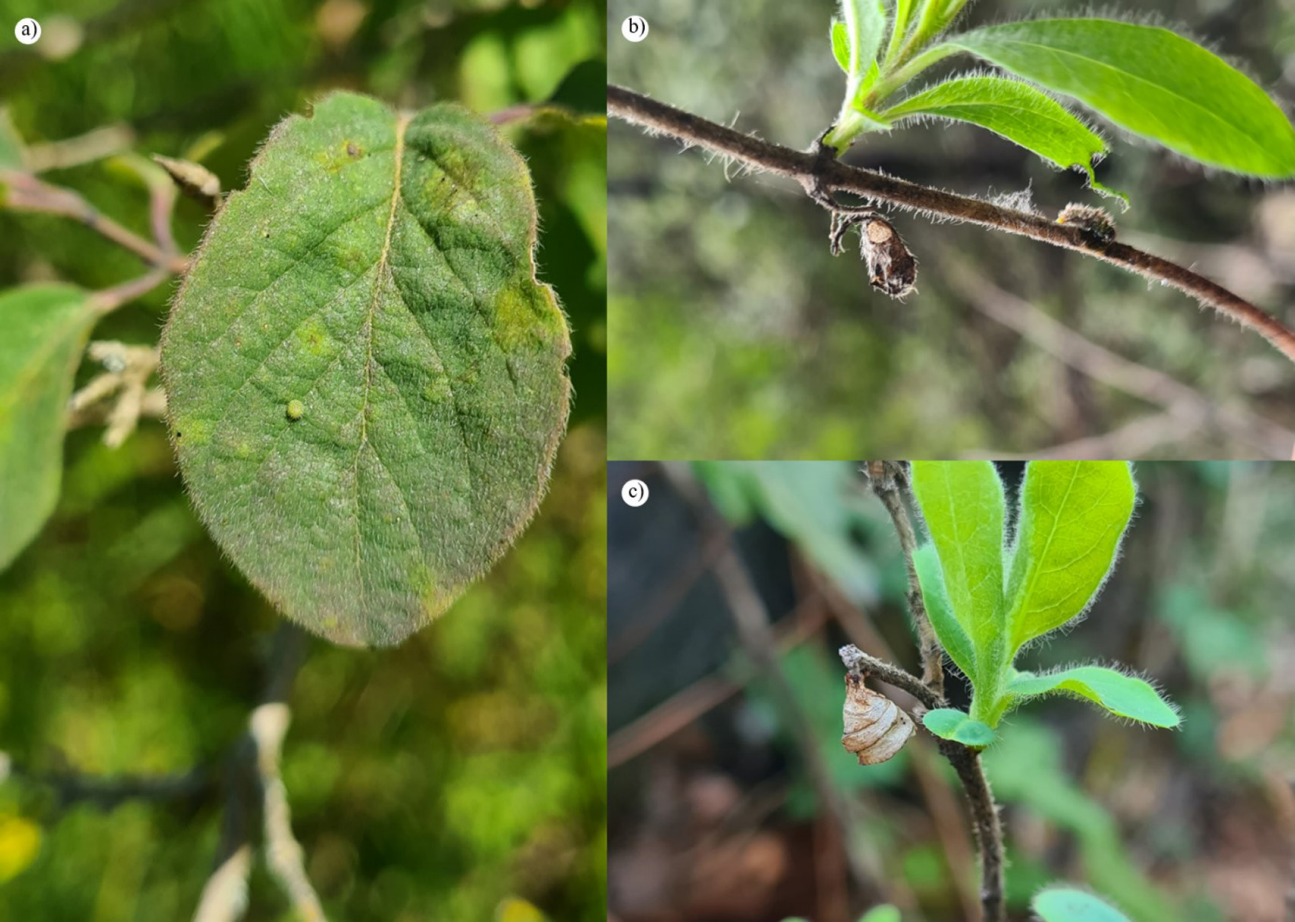
Egg of *Limenitis reducta* on *Lonicera xylosteum* (Swabian Jura, July 2025) (a), hibernaculum and larva of *Limenitis reducta* on *Lonicera etrusca* (Turkish Aegean region, March 2025) (b) and pupal remain of *Limenits reducta* on *Lonicera etrusca* (Turkish Aegea n region, March 2025) (c).

Data were collected in the Swabian Jura during several field campaigns between 2020 and 2025 and from the Turkish Aegean region in March 2025. In the Swabian Jura, we predominantly surveyed *Lonicera* plants on forest clear‐cuts, windthrows, and along forest edges, because prior observations suggested that *Limenitis reducta* is tightly linked to these habitat types (Hermann 2007). All accessible host plants within these habitats were examined for eggs, larvae, and hibernacula of *Limenitis reducta*. In contrast, in the Turkish Aegean region, all *Lonicera* plants that were visible and accessible from the main roads/paths within the study areas were surveyed. Each investigated *Lonicera* plant was examined for larvae, hibernacula, or pupal remains of *Limenitis reducta* (Figure 3).

The recorded habitat parameters per host plant included host plant height, solar radiation duration, interception and exposition. Host plant exposition was recorded in the field in the Turkish Aegean region, while it was derived from a digital elevation model of 10 m spatial resolution for the host plants in the Swabian Jura. Slope angles of less than 3° were considered as ‘flat’. For each record of *Limenitis reducta* we measured the height above ground using a metre stick.

### 2.3 Statistical Analyses

Differences in the species' host plant preferences between the core range and the range edge were analyzed using generalized linear mixed models (GLMMs). After a detailed inspection of parameter collinearity (correlograms, variance inflation factors), numerical predictor variables were standardized (z‐transformed) within each study region. We then constructed four separate GLMMs to compare the species' responses to the recorded host plant and habitat parameters between the Swabian Jura and the Turkish Aegean region. All models assumed a binomial residual distribution and a logit link function. Separate models were used because the number of records differed between predictor variables (Table 1).

**TABLE 1:** Sample sizes (number of surveyed *Lonicera* plants with and without record of *Limenitis reducta*) per habitat parameter and region/study site.

Region/study site	Solar radiation duration	Interception	Exposition	Host plant height	Record height
Occupied host plants					
Turkish Aegean region	63	63	63	63	85
Dilek Mountains	23	23	23	23	31
Sipylos Mountains	13	13	13	13	20
Ida Mountains	27	27	27	27	34
Swabian Jura	125	117	260	117	367
Allmendingen	31	28	53	28	72
Merklingen	47	47	0	47	0
Schelklingen	47	42	207	42	295
Unoccupied host plants					
Turkish Aegean region	488	488	488	488	—
Dilek Mountains	155	155	155	155	—
Sipylos Mountains	174	174	174	174	—
Ida Mountains	159	159	159	159	—
Swabian Jura	343	103	271	103	—
Allmendingen	62	24	42	24	—
Merklingen	41	41	0	41	—
Schelklingen	240	38	229	38	—
Total no. of observations	1019	771	1082	771	452

In each of the models, the predictor variable was tested in interaction with the study region (core vs. edge). Study site, nested within study region, was added to the models as an explanatory random factor variable to account for possible similarities between spatially clustered observations and thus avoid pseudoreplication. The GLMMs used the glmmTMB package (Brooks et al. 2017) in conjunction with extensive model assessment following Santon et al. (2023). Specifically, we explored residual patterns and compared dispersion, zero‐inflation, and the distribution of observed raw data with model‐simulated data. We finally used the estimated regression slopes together with their 95% compatibility intervals to quantify effect sizes and differences between regions. Additionally, we used a Welch's *t*‐test to compare the larvae's mean heights above ground between regions.

## 3 Results

**FIGURE 4:**
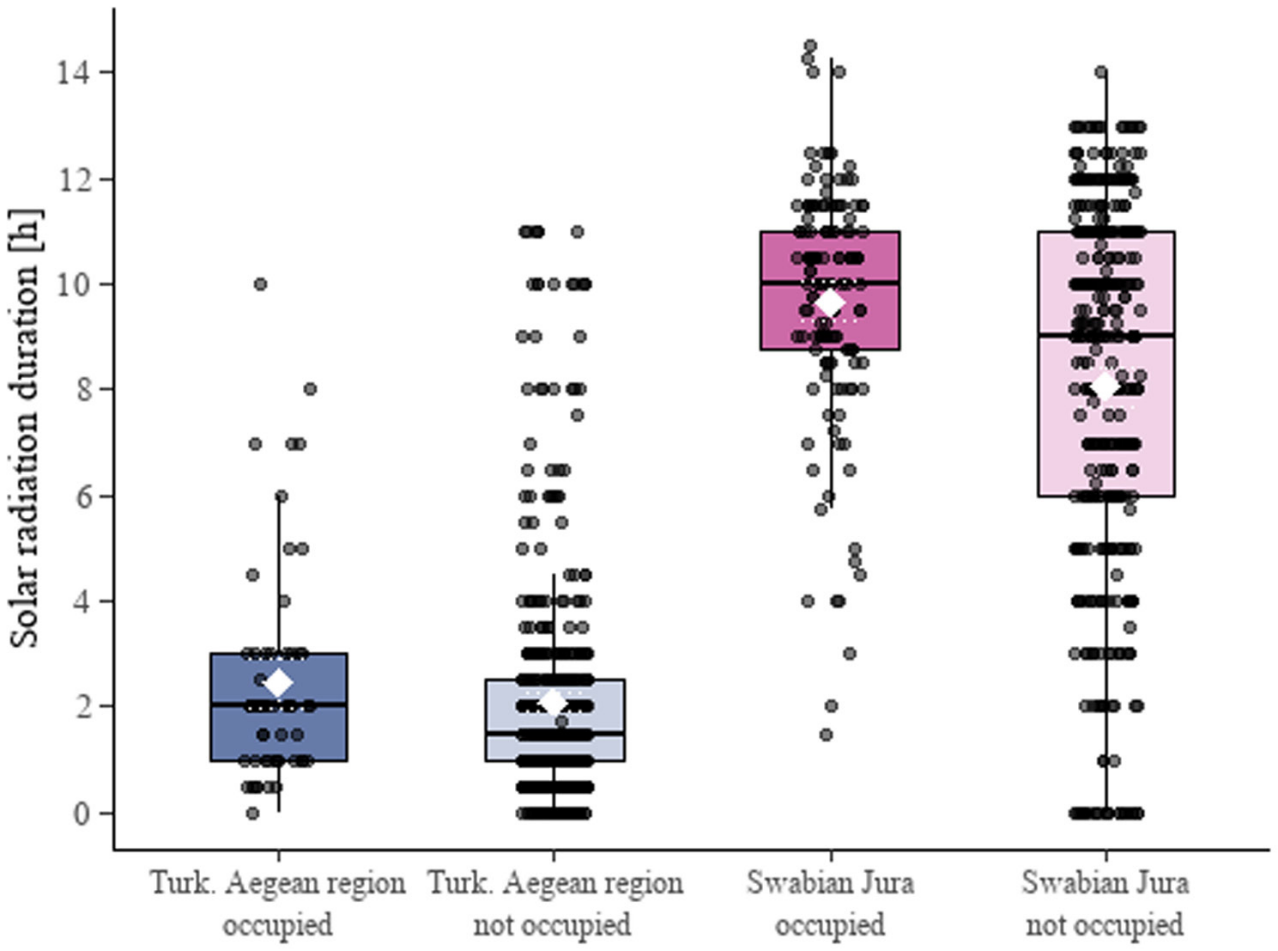
Solar radiation duration (h) for *Lonicera* plants studied in the Turkish Aegean region and the Swabian Jura. Horizontal black lines represent the median (50th percentile), boxes represent the interquartile range (25th and 75th percentile) and whiskers extend to 1.5 × interquartile range, excluding extreme values. White diamonds represent the mean and dotted white lines represent the 95% confidence interval of the mean.

**FIGURE 5:**
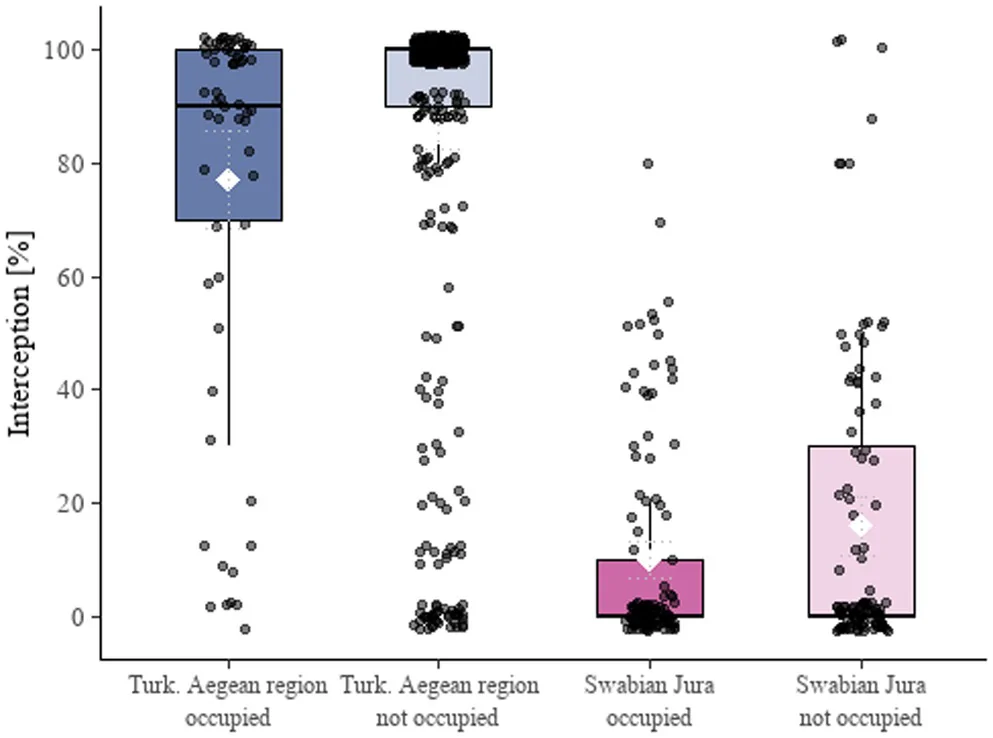
Interception (%) of *Lonicera* plants studied in the Turkish Aegean region and the Swabian Jura. Horizontal black lines represent the median (50th percentile), boxes represent the interquartile range (25th and 75th percentile) and whiskers extend to 1.5 × interquartile range, excluding extreme values. White diamonds represent the mean and dotted white lines represent the 95% confidence interval of the mean.

**FIGURE 6:**
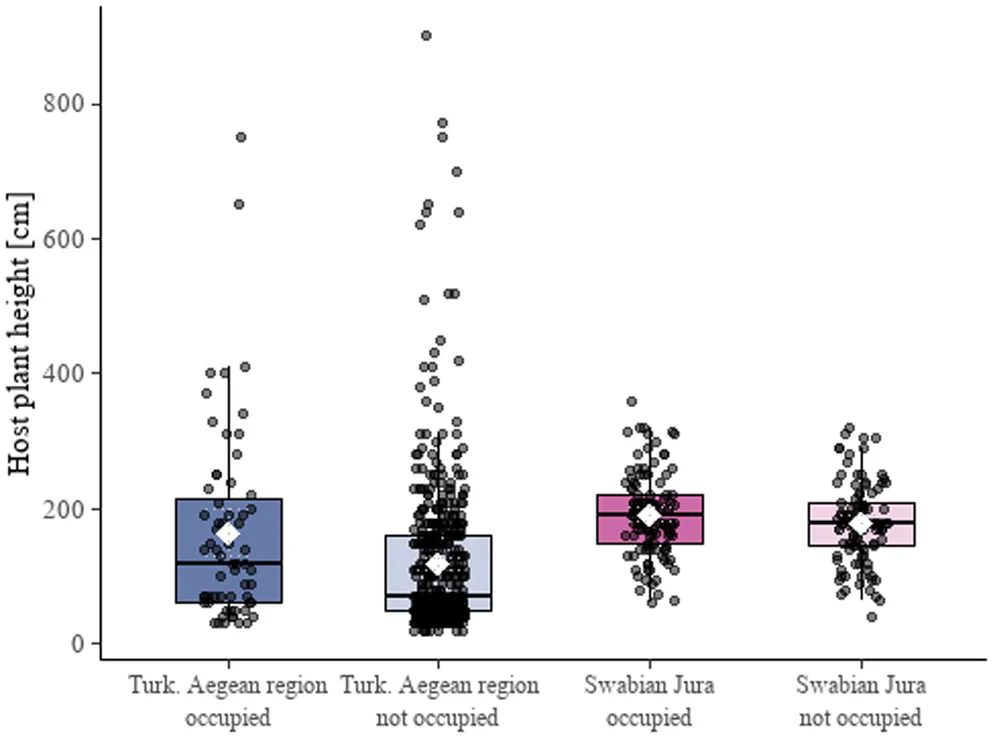
Host plant height (cm) of *Lonicera* plants studied in the Turkish Aegean region and the Swabian Jura. Horizontal black lines represent the median (50th percentile), boxes represent the interquartile range (25th and 75th percentile) and whiskers extend to 1.5 × interquartile range, excluding extreme values. White diamonds represent the mean and dotted white lines represent the 95% confidence interval of the mean.

**FIGURE 7:**
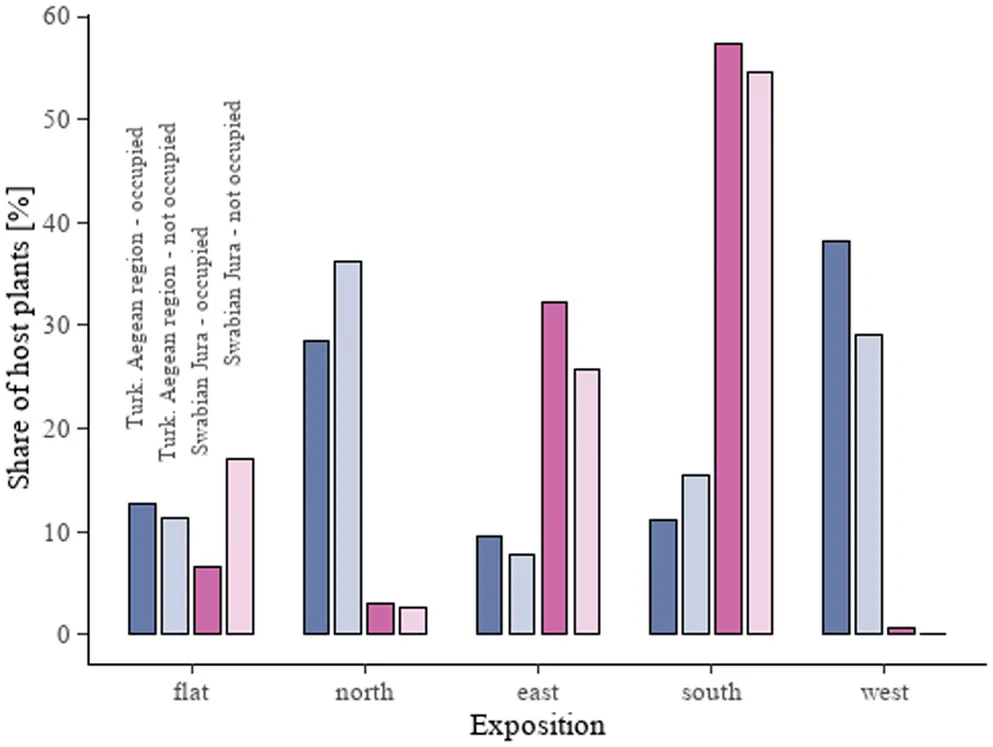
Share of host plants (%) by exposition of *Lonicera* plants studied in the Turkish Aegean region and the Swabian Jura.

**FIGURE 8:**
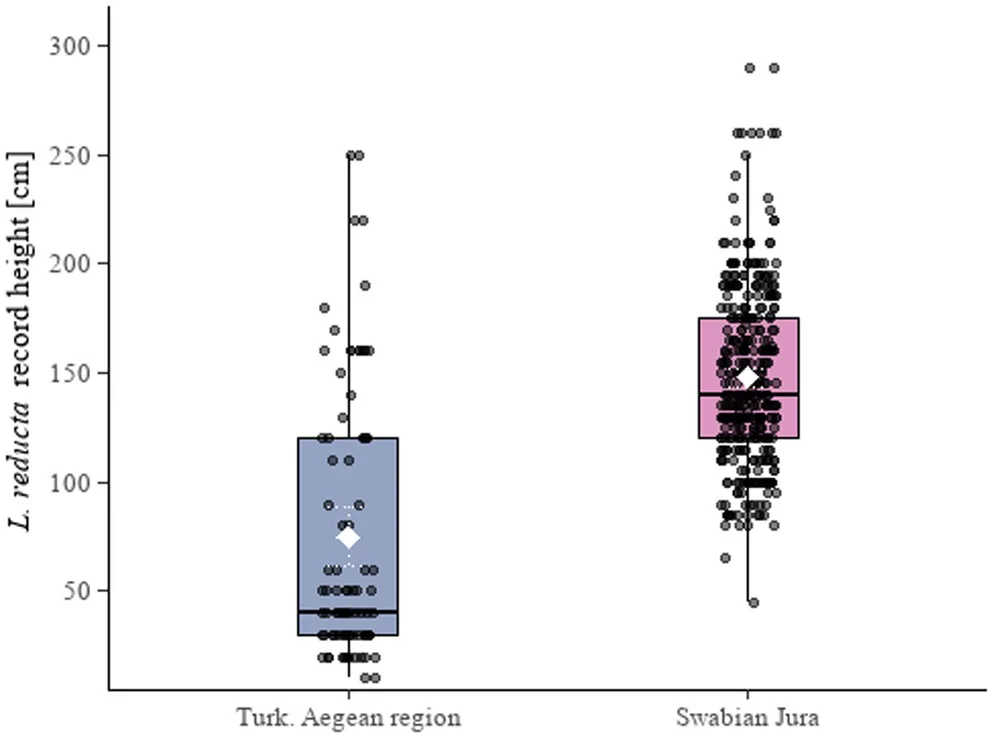
*Limenitis reducta* record height (cm) on *Lonicera* plants studied in the Turkish Aegean region and the Swabian Jura. Horizontal black lines represent the median (50th percentile), boxes represent the interquartile range (25th and 75th percentile) and whiskers extend to 1.5 × interquartile range, excluding extreme values. White diamonds represent the mean and dotted white lines represent the 95% confidence interval of the mean.

The analyses revealed considerable differences in the habitat use of *Limenitis reducta* between the two study regions. *Lonicera xylosteum* was the only available *Lonicera* species in the Swabian Jura. In contrast, three different *Lonicera* species occurred in the Turkish Aegean region and were occupied by *Limenitis reducta*. *Lonicera etrusca* accounted for 96.91% of all available *Lonicera* plants and 97.64% of *Limenitis reducta* records in the Turkish Aegean region, while *Lonicera implexa* and *Lonicera japonica* accounted for 2.36%/1.18% and 0.73%/1.18%, respectively. In the Swabian Jura, we observed a strong association between the species' presence and the daily duration of direct sunlight, whereas in the Turkish Aegean region, host plants under variable solar radiation conditions were used (Figure 4; Table 2). Likewise, the probability of *Limenitis reducta* occurrence tended to decrease with increasing sunlight interception by neighboring trees in both regions, with the effect again being slightly more pronounced in the Swabian Jura (Figure 5; Table 2). Host plant occupancy was positively associated with plant height in both regions, particularly in the Turkish Aegean region (Figure 6; Table 2). In the Swabian Jura, host plant occupancy was associated with site exposition. Host plants on east‐ and south‐facing slopes were more likely to be occupied than host plants on flat terrain. In contrast, in the Turkish Aegean region, we only observed a weak tendency for *Limenitis reducta* to select host plants on westerly‐facing slopes more often than host plants at other slope orientations (Figure 7; Table 3). The average hibernaculum height above ground differed significantly between the Swabian Jura and the Turkish Aegean region, with mean heights of 0.75 and 1.48 m, respectively (Figure 8; Welch's *t*‐test: *t* = −10.41, df = 100.71, *p* < 0.001).

**TABLE 2:** Effect sizes (changes of log‐odds per unit standard deviation).

Region	Slope mean	Lower 95% CI	Upper 95% CI
Solar radiation duration			
Turkish Aegean region	0.184	−0.070	0.438
Swabian Jura	**0.497**	0.234	0.760
Interception			
Turkish Aegean region	−0.240	−0.480	0.001
Swabian Jura	−0.273	−0.550	0.003
Host plant height			
Turkish Aegean region	**0.300**	0.089	0.510
Swabian Jura	0.244	−0.043	0.530

*Note:* Slope estimates with 95% compatibility intervals excluding zero are shown in bold.

**TABLE 3:** Pairwise comparisons of odds‐ratios among site exposition levels.

Region	Flat—E	Flat—S	Flat—W	Flat—N	W–N
Turkish Aegean region	0.921 (0.296–2.870)	1.579 (0.541–4.614)	0.861 (0.365–2.031)	1.430 (0.590–3.469)	1.662 (0.868–3.183)
Swabian Jura	**0.308** (0.162–0.584)	**0.367** (0.201–0.700)	0.000 (0.000–∞)	0.323 (0.102–1.028)	0.000 (0.000–∞)

Region	E–S	E–W	E–N	S–W	S–N
Turkish Aegean region	1.714 (0.539–5.457)	0.934 (0.356–2.449)	1.553 (0.578–4.171)	0.545 (0.225–1.323)	0.906 (0.363–2.258)
Swabian Jura	1.192 (0.807–1.761)	0.00 (0.000—∞)	1.050 (0.363–3.039)	0.000 (0.000—∞)	0.881 (0.311–2.491)

*Note:* Numbers in bold indicate significant differences, that is, when the 95% compatibility interval of the odds‐ratio did not contain 1.

## 4 Discussion

Our comparative study of habitat use in *Limenitis reducta* demonstrated that larval habitat use is context‐dependent and differs across the species' range, likely in association with different macroclimatic conditions.

All available *Lonicera* species were used as host plants in the two study regions. At the northern range edge, the Swabian Jura, *Lonicera xylosteum* was the only available host plant species. In the Turkish Aegean region, however, larvae were found on the dominant *Lonicera etrusca* as well as on the less common *Lonicera implexa* and the rare, non‐native *Lonicera japonica* . This broader host plant spectrum in the Mediterranean region is consistent with earlier observations from southern Europe and Anatolia reporting regionally variable use of several *Lonicera species* (Stefanescu and Jubany 2003; Hesselbarth et al. 1995). Our data suggest that *Lonicera etrusca* is quantitatively the most important host plant in the Turkish Aegean region but is not preferred over other *Lonicera* species. The acceptance of various *Lonicera* species across the distribution range of *Limenitis reducta* underscores a degree of ecological flexibility within a phylogenetically defined host use.

In addition to regional differences in host plant species, we detected different microhabitat associations between the two study regions. Solar radiation was more strongly associated with host plant occupancy at the northern range edge than in the Turkish Aegean region. This pattern is notable because our sampling in the Swabian Jura was restricted to generally well‐lit habitats such as windthrows and clear‐cuts, thereby reducing the contrast in solar radiation among sampled host plants. Host plants with a solar radiation duration of less than 7 h per day were used in only very few instances in the Swabian Jura. In contrast, many species records were made under such solar radiation conditions in the Turkish Aegean region. At the Turkish study sites *Limenitis reducta* was found under almost all available solar radiation conditions. The broad range of occupied microhabitats in the Turkish Aegean region might suggest opportunistic habitat use. Previous studies have reported broader and more opportunistic microhabitat use in newly established edge populations of *Lycaena dispar* during range expansion (Martin et al. 2021). However, this was not the case for *Limenitis reducta* in the Turkish Aegean region. The much more specific habitat associations at the northern range edge could be explained by stronger thermal constraints than in the Mediterranean populations. This interpretation is consistent with expectations that populations near climatic range margins may become increasingly associated with a restricted subset of suitable microhabitats (Oliver et al. 2012). A clear relationship between *Limenitis reducta* vital rates and host plant solar radiation in the Swabian Jura was shown in a previous study (Hinneberg et al. 2025), indicating that the average temperatures in this region are probably below the thermal optimum of *Limenitis reducta* larvae. Although we propose thermal constraints of the larvae as the major driver of strong differences in *Limenitis reducta* microhabitat selection between Central Europe and the Mediterranean region, regional differences in vegetation structure, disturbance history, and habitat availability may also contribute to the observed pattern. Because only one Central European and one Mediterranean study region were compared in our study, we cannot exclude the possibility that some of the observed patterns may reflect characteristics of the specific regions rather than general differences in habitat use between Central European and Mediterranean populations.

Host plant height was positively associated with occupancy in both regions, especially in the Turkish Aegean region. Similar patterns have been reported for other nymphalid butterflies, where larger host plants were preferred for oviposition (Anthes et al. 2003; Johansson et al. 2024). Larger shrubs likely provide greater resource availability and structural heterogeneity, increasing the likelihood that females find suitable positions for egg laying within the host plant. In the Turkish Aegean region, larvae were predominantly found in the lower parts of the host plants, possibly due to more stable microclimatic conditions near the ground. Stuhldreher and Fartmann (2018) have shown that temperature and humidity can differ substantially over very fine spatial scales and that vegetation structure and the near‐ground boundary layer can strongly influence microclimatic conditions. In contrast, higher larval positions may increase sun exposure and thus daytime warming (Hinneberg et al. 2025). This solar radiation‐induced increase in local temperature may be particularly important for larval development under cooler macroclimatic conditions, that is, at the northern range edge. Thus, differences in the vertical positioning of eggs and larval stages might be an adaptive response to different macroclimatic conditions within the species' distribution range.

We believe that the different patterns of habitat use observed in our study provide an example of climatic niche conservatism (Wiens et al. 2010), which is realized by the use of distinct microhabitats of different sun exposure under contrasting macroclimatic conditions. However, we acknowledge that detailed recordings of air temperature at the host plants would be necessary to validate our hypothesis. The stronger association of the northern population with highly sun‐exposed host plants compared with the much broader range of occupied microhabitats in the Mediterranean study region indicates a reduction in realized microhabitat niche breadth at the northern range margin. Oliver et al. (2009) described the same general pattern across 41 butterfly species, showing that habitat associations become increasingly restricted toward climatically unsuitable parts of species' distributions. Hällfors et al. (2024) proposed that organisms with narrower climatic tolerances depend more strongly on suitable environmental conditions, whereas broader tolerances permit greater ecological flexibility. Fine‐scale microclimatic heterogeneity may provide the local thermal conditions required to fulfill these climatic requirements (Suggitt et al. 2018), while the narrower climatic niches observed in butterfly species occupying climatic extremes (Klečková et al. 2023) further support the expectation of increased ecological specialization under unsuitable climatic conditions.

Our results demonstrate that ecological inference based on studies from a single region may fail to capture the full extent of habitat associations across a species' geographic distribution. However, our conclusions are based on comparisons between one northern and one Mediterranean study region. Consequently, the observed differences should not be interpreted as universally representative of all Central European and Mediterranean populations of *Limenitis reducta*. Replication across additional regions throughout the species' distribution will be necessary to determine whether the observed habitat associations reflect general geographic patterns or partly result from regional environmental particularities. For applied species conservation, our findings imply that habitat management strategies derived from populations in one part of the species' range should not be applied uncritically to other regions. Effective conservation of *Limenitis reducta* is therefore likely to benefit from region‐specific management approaches that account for regional climatic contexts as well as associated differences in forest structure and host plant availability. Local management will nevertheless benefit from a better understanding of habitat associations across the species' distribution range, in particular for adapting habitat management strategies under future climate change.

## Author Contributions

**Julius Kaiser:** conceptualization (equal), funding acquisition (equal). **Heiko Hinneberg:** data curation (equal), formal analysis (equal), funding acquisition (equal). **Thomas K. Gottschalk:** conceptualization (equal), data curation (equal).

## Conflicts of Interest

The authors declare no conflicts of interest.

## Data Availability

The data that support the findings of this study are openly available in Dryad at https://doi.org/10.5061/dryad.qz612jmx6.
